# Factors associated with the discontinuation of hormonal contraceptives in women of Lima, Peru

**DOI:** 10.18332/ejm/174478

**Published:** 2024-01-05

**Authors:** Lesly Cruz-Lama, Robin Villalobos, Mercedes Tello, Jhony A. De La Cruz-Vargas, Ericson L. Gutierrez

**Affiliations:** 1 Instituto de Investigaciones en Ciencias Biomédicas, Universidad Ricardo Palma, Lima, Peru; 2 Facultad de Medicina, Universidad Nacional Mayor de San Marcos, Lima, Peru; 3 Ministerio de Salud, Dirección de Redes Integradas de Salud Lima Sur, Lima, Peru

**Keywords:** family planning, treatment adherence and compliance, contraceptive agents

## Abstract

**INTRODUCTION:**

Family planning is a right and a tool that offers the possibility of choosing how many children to have. Its importance lies in the possibility of avoiding an unwanted pregnancy and its consequences. Our objective was to determine the factors associated with discontinuing hormonal contraceptives in women of childbearing age who attended the La Libertad Health Center in January 2023.

**METHODS:**

The study was observational, analytical, and cross-sectional. A total of 100 women of childbearing age who were users of hormonal contraceptives were included. Descriptive statistics were performed, frequency measurements and measures of central tendency were calculated, bivariate statistics were performed and the prevalence ratio (PR) was calculated, and a robust Poisson regression model was performed to assess the associated independent factors. All calculations were made with a confidence level of 95%.

**RESULTS:**

The educational level (PR=1.74; 95% CI: 1.22–2.48, p=0.006), the distance to the health center (PR=7.32; 95% CI: 1.1–48.5, p=0.001), having presented adverse events (PR=26.38; 95% CI: 3.8–183, p=0.001), and that the health staff had not identified the need for contraception (PR=3.12; 95% CI: 0.87–11.10, p=0.01) were associated with stopping a hormonal contraceptive. After introducing the variables to the regression model, the only independently associated factor was having presented an adverse event with the use of hormonal contraceptives (adjusted prevalence ratio, APR=3.33; 95% CI: 2.1–5.2, p<0.001).

**CONCLUSIONS:**

In this population, the factors associated with the discontinuation of hormonal contraceptives were education level, distance to the health center, having presented some adverse event with its use, and that health staff had not identified the need for contraception. The only independently associated factor was having presented an adverse event.

## INTRODUCTION

In 1968, the United Nations International Conference established that family planning was a right based on individuals being able to decide how many children to have^1-3^. According to the World Health Organization, in 2019, out of 1.9 billion women of reproductive age worldwide, 842 million used some kind of contraceptive method. However, 270 million people still have contraceptive-related needs that are not met^4^. In Peru, one in three women uses a traditional contraceptive method, while around half (54.5%) use a modern contraceptive method, a rate that is very much below the average regional (70%). On the other hand, the Demographic and Family Health Survey highlights that 63.8% of women with a stable partner did not wish to have any more pregnancies^5,6^. Some consequences of an unplanned pregnancy, especially in a vulnerable population such as adolescents, are dropping out of school and dedicating themselves to unpaid domestic work such as taking care of dependent people and housework^7-10^. Likewise, according to the World Health Organization (WHO) and the Guttmacher Institute, between 2010 and 2014, there were 25 million unsafe abortions, of which 97% were in developing countries, distributed among the continents of Africa, Asia, and Latin America. In Peru, the cost of complicated and uncomplicated abortions demanded a significant cost for hospitals and the women themselves. Misinformation in family planning matters, brings with it unwanted maternity or paternity, which leads to consequences for the parents as well as children in the short- and long-term. For this reason, one of the key factors to consider in contraception is adherence to the method, understood within the context that when a woman does not wish to get pregnant, she must continue the contraceptive method that she chose. According to the aforementioned, our objective is to learn the factors associated with the abandonment of contraceptives in an urban population in Lima.

## METHODS

### Study design

In an observational, analytical cross-sectional study, data were obtained through a survey that collected associated factors (institutional, sociocultural, and personal determinants) and their association with the abandonment of contraceptives.

### Participants

The study participants were women of reproductive age who attended La Libertad Health Center, located in the district of San Juan de Lurigancho (Lima, Peru), in January 2023. The sample size was calculated with the EPIDAT 4.2 statistical program. The formula for comparing independent rates was used; the proportion of women (68.8%) who gained weight and abandoned the hormonal contraceptives (HCs) was assumed as group 1, and as group 2, the proportion of women (37.5%) who did not gain weight and abandoned the hormonal contraceptives. A power of 90% and a confidence level of 95% was used, and exposed to non-exposed ratio was 1:1, taken from a previous study. We use this variable to obtain the sample number because, according to our literature search, it is one of the most important for the abandonment of HCs.

Lastly, the minimum sample size was 100 participants. A systematic sampling with a random start was performed. A random number between 1 and 3 was chosen; later, patients were interviewed at intervals of 3 every time they left the doctor’s office. Women of fertile age aged 18–45 years who were using HCs or who abandoned them over 30 days, were included. Patients from other health centers or women who did not know how to read and write, and who did not wish to participate in the study, were excluded.

### Ethical aspects

Ethical approval was obtained from the Ethics and Investigation Committee of the Medical School at Ricardo Palma University. We requested informed consent from the participants, after providing detailed information about the study that was being carried out; the consent was obtained upon leaving the offices and before applying the instrument. Likewise, we were granted authorization by the Head of La Libertad Health Center to carry out the study. The information obtained in this study is confidential (the identity of the participants is undisclosed).

### Variables

For the variable of interest, we categorized the answers to the question: ‘Did you interrupt the use of hormonal contraceptives for a period greater than 30 days?’, with the answer ‘yes’ (discontinuation of contraceptives) or ‘no’ (no discontinuation of contraceptives). The following independent variables were also included: institutional factors (user knowledge, distance to the health center, drug availability, and quality of care), sociocultural factors (religion, education level, financial resources, unemployment, family, couple, or partner influence), and personal factors (self-care, shyness, fear, and time).

### Procedures

We requested approval from the authorities of the health center, and then we located the women in the medicine and obstetrics departments. We carried out the survey, which was self-administered by the women who met the inclusion criteria. The instrument was previously applied to a similar population to that of the present study, which came from the same health jurisdiction. It was validated by expert judgment and a pilot test, showing high concordance; likewise, it showed adequate psychometrics. The value of Cronbach’s alpha was 0.71.

### Statistical analysis

We used the SPSS version 27 statistical package to enter the database and perform statistical analysis. A descriptive analysis with quantitative variables was performed. Afterwards, a bivariate analysis was carried out to determine the association between the independent (associated factors) and dependent variables (discontinuation of hormonal contraceptives). A chi-squared test was used for the categorical variables. The prevalence ratio was calculated to establish the association. Finally, the variables that were significant in the bivariate analysis were entered into the robust Poisson regression model. Significance was set at p<0.05. All the calculations were carried out at a confidence level of 95%.

## RESULTS

In this study, we included 100 participants, and 50% of them responded that they had abandoned the HCs; 52% of the women were aged ≤30 years. Only 26% had a higher education level, whether technical or college. Most (80%) women were married or living with a partner. The majority were unemployed (69%), including housewives and students, and 58% of participants declared they were Catholic. An association was found between low education level and abandonment of HCs (PR=1.74; 95% CI: 1.22–2.48, p=0.006) (Table 1). Regarding personal factors, 61% deemed they had enough knowledge regarding HCs, yet 56% discontinued it. Of those who felt embarrassed to ask about HCs, 56% also discontinued them. Regarding the relationship between participants’ personal factors and discontinuation of hormonal contraceptive methods, we found an association between having an adverse effect (PR=26.38; 95% CI: 3.8–183, p=0.001) and discontinuation of HCs (Table 2). Regarding institutional factors, 92% had knowledge of the functioning of a family planning center; of these, 90% discontinued it. However, 8% did not know of the existence of a family planning service and received orientation outside of the center; of these, 10% discontinued it. We found two relevant associations between the distance to the health center (PR=7.32; 95% CI: 1.1–48.5, p=0.001) and the health staff not identifying the need to use contraceptive methods (PR=3.12; 95% CI: 0.87–11.10, p=0.01) and the discontinuation of HCs (Table 3). Regarding the sociocultural factors, out of the group that deemed it was good to provide information about this topic in schools (93%), 90% discontinued it. In all, 7% of the remainder thought it was counterproductive to provide guidance in schools, of which 10% discontinued it. Furthermore, of the 78% that used some kind of communication method to seek orientation on the topic, 76% discontinued it. On the other hand, 17% believed that using a contraception method was a sin; of these, 78% discontinued it. It was reported by 93% that their religion allowed them to use a family planning method. Finally, from the relation between the participant’s sociocultural factors and discontinuation of hormonal contraceptives, we did not find any association between these variables (Table 4). After carrying out the multivariate analysis, among the associated variables in the bivariate analysis, the only independent variable associated with abandonment of HCs was having a prior adverse event (APR=3.33; 95% CI: 2.1–5.2, p <0.001).

**Table 1:** Relationship between the sociodemographics characteristics and abandonment of hormonal contraceptive methods in women of Lima, Peru, 2023 (N=100)

*Variables*	*n*	*%*	*Interrupted HCs*				*PR (95% CI)*	*p*
			*Yes*		*No*			
			*n*	*%*	*n*	*%*		
**Age** (years)								
≤30	52	52	25	50	27	54	0.92 (0.62–1.36)	0.68
>30	48	48	25	50	23	46		
**Education level**								
Low	74	74	43	86	31	62	1.74 (1.22–2.48)	0.006
High	26	26	7	14	19	38		
**Marital status**								
Single/separated/widowed	20	20	13	26	7	14		0.134
Living with a partner or married	80	80	37	74	43	86	0.65 (0.34–1.22)	
**Work**								
Paid work	31	31	16	32	15	30		0.829
Unpaid work	69	69	34	68	35	70	0.95 (0.62–1.46)	
**Religion**								
Catholic	58	58	30	60	28	56		0.685
Other	42	42	20	40	22	44	0.92(0.62–1.36)	
**Number of sexual partners**								
1	39	39	24	48	15	30		0.065
≥1	61	61	26	52	35	70	0.67 (0.42–1.05)	
**Age of onset of sexual activity** (years)								
≥18	49	49	32	64	27	54	0.81 (0.55–1.20)	0.309
<18	41	41	18	36	23	46		
**Abortions**								
None	71	71	38	76	33	66		0.271
≥1	29	29	12	24	17	34	0.79 (0.53–1.17)	
**Living children**								
1	44	44	21	42	23	46		0.687
>1	56	56	29	58	27	54	1.08 (0.73–1.60)	

* HCs: hormonal contraceptives.

**Table 2:** Relationship between personal factors and abandonment of hormonal contraceptive methods in women of Lima, Peru, 2023 (N=100)

*Variables*	*n*	*%*	*Interrupted HCs*				*PR (95% CI)*	*p*
			*Yes*		*No*			
			*n*	*%*	*n*	*%*		
**Deemed having enough knowledge about HCM**								
Yes	61	61	28	56	33	66		0.3
No	39	39	22	44	17	34	1.24 (0.81–1.89)	
**Embarrassed to ask about HCM**								
Yes	50	50	28	56	22	44	1.27 (0.85–1.89)	0.23
No	50	50	22	44	28	56		
**Had some adverse effect**								
Yes	35	35	34	32	1	2	26.38 (3.8–183)	0.001
No	65	65	16	68	49	98		
**Had enough time to use a HCM**								
Yes	73	73	34	68	39	78		0.26
No	27	27	16	32	11	22	1.31 (0.79–2.16)	
**Deemed that it is important to use a HCM**								
Yes	93	93	45	90	48	96		0.46
No	7	7	5	10	2	4	1.8 (0.55–5.92)	
**Deemed it important to plan how many children you wish to have**								
Yes	96	96	47	94	49	98		0.61
No	4	4	3	6	1	2	2.04 (0.37–11.27)	

* HCs: Hormonal contraceptives

**Table 3:** Relationship between institutional factors and abandonment of hormonal contraceptive methods in women of Lima, Peru, 2023 (N=100)

*Variables*	*n*	*%*	*Interrupted HCs*				*PR (95% CI)*	*p*
			*Yes*		*No*			
			*n*	*%*	*n*	*%*		
**Aware that the health center offered free HCs**								
Yes	92	92	45	90	47	94		0.71
No	8	8	5	10	3	6	1.36 (0.54–3.40)	
**Aware that the health center was a planning center**								
Yes	92	92	45	90	47	94		0.71
No	8	8	5	10	3	6	1.36 (0.54–3.40)	
**Aware that the family planning office was available Monday to Saturday during morning and afternoon shifts**								
Yes	81	81	38	76	43	86		0.2
No	19	19	12	24	7	14	1.44 (0.77–2.68)	
**The family planning methods of the Health Center La Libertad satisfy your needs**								
Yes	79	92.9	37	90.3	42	95.5		0.42
No	6	7.1	4	9.7	2	4.5	1.59 (0.50–5.03)	
**According to the service received in the family planning office by the staff that treated you, do you consider that:**								
**The health staff were discreet and reliable^a^**								
Yes	77	90.6	37	90.2	40	90.9		1
No	8	9.4	4	9.8	4	9.1	1.03 (0.50- 2.14)	
**The staff identified your need to use some contraceptive method^a^**								
Yes	74	87.1	32	78.1	42	95.5		0.01
No	11	12.9	9	21.9	2	4.5	3.12 (0.87–11.10)	
**The health staff responded satisfactorily to your needs or doubts^a^**								
Yes	75	88.2	34	82.9	41	93.2		0.18
No	10	11.8	7	17.1	3	6.8	1.82 (0.69–4.80)	
**The health staff verified that you had understood all the information pertaining to the same^a^**								
Yes	68	80	33	80.5	35	79.5		0.91
No	17	20	8	19.5	9	20.5	0.97 (0.58–1.61)	
**The health staff kept a cordial and respectful attitude towards you^a^**								
Yes	80	94.1	37	90.2	43	97.8		0.19
No	5	5.9	4	9.8	1	2.2	1.79 (0.46–15.69)	
**You believe that the distance from your home to the health center was a determinant not go to the health center to pick up your contraceptive method**								
Yes	13	13	12	24	1	2	7.32 (1.1–48.5)	0.001
No	87	87	38	76	49	98		

* HCs: hormonal contraceptives,

a N=85.

**Table 4:** Relationship between sociocultural factors and abandonment of hormonal contraceptive methods in women of Lima, Peru, 2023 (N=100)

*Variables*	*n*	*%*	*Interrupted the use of HCs*				*PR (95% CI)*	*p*
			*Yes*		*No*			
			*n*	*%*	*n*	*%*		
**Do you believe it is good that schools provide information about contraceptive methods?**								
Yes	93	93	45	90	48	96		0.43
No	7	7	5	10	2	4	1.8 (0.55–5.92)	
**Within your family circle, were contraceptive methods ever mentioned?**								
Yes	41	41	22	44	19	38		0.54
No	59	59	28	56	31	62	0.88 (0.58–1.32)	
**Do you believe that the information provided in your location about contraceptive methods is adequate?**								
Yes	62	62	33	66	29	58		0.41
No	38	38	17	34	21	42	0.84 (0.57–1.25)	
**Did you ever use any form of communication to learn about contraceptive methods?**								
Yes	78	78	38	76	40	80		0.62
No	22	22	12	24	10	20	1.12 (0.68–1.87)	
**Did you ever think that the use of contraceptive methods was a sin?**								
Yes	17	17	39	78	44	88	1.5 (0.76–2.94)	0.18
No	83	83	11	22	6	12		
**Does your religion allows you to use contraceptive methods**								
Yes	93	93	45	90	48	96		0.43
No	7	7	5	10	2	4	1.8 (0.55–5.92)	
**You believe your partner has a right to forbid you to use contraceptive methods**								
Yes	7	7	4	8	3	6	1.17 (0.49–2.83)	1
No	93	93	46	92	47	94		
**Did you ever believe that a women should not plan, since her duty is to give her husband children?**								
Yes	3	3	2	4	1	18	1.51 (0.3–7.6)	1
No	97	97	48	96	49	82		
**Did you have enough transportation and economic resources to attend your appointments at the Family Planning center?**								
Yes	73	73	35	70	38	76		0.49
No	27	27	15	30	12	24	1.17 (0.72–1.88)	

* HCs: Hormonal contraceptives

**Table 5:** Factors independently associated with discontinuation of hormonal contraceptives in women of Lima, Peru, 2023 (N=100)

*Independent variables*	*APR (95% CI)*	*p*
Educational level	0.6 ( 0.33–1.08)	0.09
Had some adverse event	3.33 (2.1–5.28)	<0.001
The staff identified your need to use some form of contraceptive method	1.03 (0.78–1.37)	0.79
You believe that the distance from your home to the health center was a determinant not to pick up your contraceptive method at the health center	1.29 ( 0.95–1.76)	0.09

APR: adjusted prevalence ratio.

## DISCUSSION

In our unadjusted analyses, the factors associated with the abandonment of hormonal contraceptive methods were education level, distance to the health center, having presented some adverse event with its use, and that health staff had not identified the need for contraception. An independent factor in the adjusted analyses was having presented an adverse event. Regarding adverse effects as a cause for abandonment of HCM, similar results were found in other research^11-13^. These authors indicated that the most common adverse effects were headaches, weight gain, and, in the case of implants, uterine hemorrhage. Evidence also exists that an adequate explanation to the patient about possible adverse events would clarify the erroneous concepts and would then result in lower abandonment rates for HCM^14,15^.

Regarding the association between the distance between home and the health center, our results coincide with other research^16,17^, although in these studies, the population was in a rural zone as opposed to ours, which was in an urban zone. In their results, it is emphasized that the percentage of women who used modern contraceptives decreased as the distance to the health center increased.

We also found an association between low education level and abandonment of HCM. Although the study was performed in an urban zone, the capital of the country, with greater access to education, we still found women who only had elementary school completed. In prior studies^18^, it was found that those participants with some college education were less likely to report an interruption to HCs. One study^19^ obtained similar findings: women with elementary education were less likely to abandon contraception compared to those who were illiterate. This may be because participants with better education have a better understanding of what the methods consist of and their advantages and disadvantages. However, different results were found by other studies^20^, where it was found that a higher education was associated with greater interruption, which may be explained by the fact that with greater knowledge and access to information, there may be greater ease in obtaining another method. Our study showed that not identifying the need for contraception for the patient by the health staff was another variable that had an association with abandonment of HCs. This situation is understood as individualizing the care of each user and being able to offer a method. One study found that optimal first counseling is related to the use of medroxyprogesterone without interruptions.

### Limitations

One of the limitations of our research study was the small sample size; therefore, the results of this study are only representative of the population that attends a health facility. Another limitation was the nature of the study, which was cross-sectional, which did not allow us to establish a definite causal judgment due to the temporary ambiguity that happens when simultaneously collecting information. Another design disadvantage is that it provides data from a particular moment in time, which means that if the same population is examined at another time, the results obtained may be different. Finally, due to the small number of participants, estimates were obtained with a wide confidence interval. However, despite these limitations, this study addresses a relevant public health topic for Peru within the context of primary healthcare.

## CONCLUSIONS

The results of this study could be used by local authorities to apply intervention strategies to improve access to and adherence to hormonal contraceptive methods. We suggest providing advice prior to choosing a contraceptive method so that each user understands how hormonal contraceptive methods work. We also recommended a technical assistance program for health professionals who offer contraceptive methods to women, since the quality of their care is related to the proper use of HCs.

## Data Availability

The data supporting this research are available from the authors on reasonable request.
